# A Case of Potential Oral Desmopressin Malabsorption Secondary to Concurrent Tube Feed Administration in a Patient With Arginine Vasopressin Deficiency

**DOI:** 10.7759/cureus.115156

**Published:** 2026-08-25

**Authors:** Gunjan Arora, Vijaya Deepika Perugu, Andjela Drincic

**Affiliations:** 1 Endocrinology, Diabetes and Metabolism, University of Nebraska Medical Center, Omaha, USA

**Keywords:** arginine vasopressin deficiency, desmopressin (ddavp), hypernatremia, polyuria, tube feed

## Abstract

Arginine vasopressin deficiency (AVP-D) is an endocrine disorder characterized by dilute, non-osmotic polyuria and hypernatremia, caused by a deficiency of antidiuretic hormone (ADH). Drug resources indicate that food may reduce absorption of oral desmopressin (DDAVP). However, it reportedly does not affect pharmacodynamics; therefore, no precautions are currently advised regarding food intake or tube feeding (TF). We present a case in which severe polyuria and hypernatremia developed after TF was initiated in a patient receiving oral DDAVP for AVP-D, which was corrected upon switching to subcutaneous DDAVP, suggesting TF-induced enteral DDAVP malabsorption.

## Introduction

Arginine vasopressin deficiency (AVP-D) is an endocrine disorder characterized by dilute, non-osmotic polyuria and hypernatremia, caused by a deficiency of antidiuretic hormone (ADH) [1]. ADH is a hormone produced by the hypothalamus and transported by the neuronal axons to the posterior pituitary, where it is stored and released into the bloodstream physiologically in response to increased blood osmolality, decreased blood volume, and stress [1,2]. The clinical presentation of AVP-D can include polyuria (the hallmark feature of more than 3 L of dilute urine per day), polydipsia, hypernatremia, and dehydration. The excretion of a large volume of dilute urine can lead to electrolyte disturbances. Desmopressin (DDAVP), an ADH analog, is the preferred treatment for AVP-D as desmopressin repletes the deficient ADH [3-5]. Desmopressin can be administered orally, subcutaneously, and intravenously.

Drug resources indicate that food may reduce absorption of oral DDAVP. However, it reportedly does not affect its pharmacodynamics [6-8]; as such, no precautions are currently advised with food intake or tube feed (TF) administration. Here, we present a case where severe polyuria and hypernatremia developed after TF was initiated in a patient receiving oral DDAVP for AVP-D, which was corrected upon switching to subcutaneous DDAVP. This case highlights the importance of timely and effective DDAVP administration in patients with adipsic AVP-D, as they are especially vulnerable to sodium derangements due to lack of thirst, which can have devastating consequences, including changes in mental status and seizures.

This case report was previously presented at the 2025 Endocrine Society meeting in San Francisco, CA, in July 2025.

## Case presentation

A 65-year-old female with a past medical history significant for craniopharyngioma status post-resection and resultant AVP-D on oral DDAVP presented to the emergency department from a long-term care facility with altered mental status and concern for a stroke. She was found to have an acute ischemic infarct.

Before admission, the patient was on DDAVP 200 µg by mouth twice daily. She had an intact thirst mechanism along with stable sodium (Na) levels over the prior years. On admission, her sodium level was 141 mmol/L, potassium level was 3.1 mmol/L, and blood urea nitrogen/creatinine ratio was 21.3 mgUN/mgCR (Table 1). She failed the dysphagia screen and subsequently underwent percutaneous endoscopic gastrostomy (PEG) tube placement along with TF. DDAVP was switched to a subcutaneous formulation at 1 µg subcutaneously twice a day. After three days, due to clinical improvement, DDAVP 1 µg subcutaneously twice a day was switched to DDAVP 200 µg orally via a feeding tube. The next day, her clinical condition deteriorated. She developed altered mental status and severe hypernatremia (Na increased from 142 mmol/L the day prior to 176 mmol/L) with polyuria (urine output increased from 0.5-1 mL/kg/hour, normal range: 0.5-1.5 mL/kg/hour on prior days to 1.6 mL/kg/hour) necessitating admission to the intensive care unit (ICU). There was no free water restriction or other medication changes around the time of clinical deterioration to account for the hypernatremia.

**Table 1:** Laboratory findings on admission.

	Result	Reference range
Sodium	141	136–145 mmol/L
Potassium	3.1	3.5–5.1 mmol/L
Chloride	102	98–107 mmol/L
Bicarbonate	27	22–32 mmol/L
Anion gap	12	4–15 mmol/L
Glucose	138	70–139 mg/dL
Blood urea nitrogen	16	6–20 mg/dL
Creatinine	0.75	0.44–1.03 mg/dL
Blood urea nitrogen/Creatinine ratio	21.3	10–20 mgUN/mgCR
Osmolality	294	275–295 mOsm/kg
Calcium	10	8.6–10.4 mg/dL
Glomerular filtration rate	88	>59 mL/minute/1.73m^2^

Upon arrival at the ICU, DDAVP was switched back to the subcutaneous route of administration with scheduled sodium level checks every two hours, along with fluid replacement. The next day, sodium level improved to 146 mmol/L and remained stable, and her mentation subsequently improved. She was able to transfer out of the ICU after a three-day stay. Endocrinology was consulted given the complex nature of the case with strict NPO status, continuous TF administration, requirement for subcutaneous DDAVP, and sensitive sodium homeostasis. Endocrinology service evaluated and advised that the patient be discharged on DDAVP 1 µg subcutaneously twice daily. Sodium level remained within normal limits for weeks, with concentrated urine at follow-up.

## Discussion

We report a case of non-osmotic polyuria with hypernatremia in a patient with AVP-D (previously well managed on oral DDAVP dosing before admission in the absence of TF) who developed dysphagia and adipsia in the setting of acute ischemic infarct, received oral DDAVP with concurrent TF, followed by resolution of hypernatremia upon transition from oral DDAVP to subcutaneous formulation. We postulate that enteral intake (TF) caused DDAVP malabsorption.

Desmopressin can be administered intravenously, subcutaneously, orally, or intranasally [1]. Administration via intravenous and subcutaneous routes is primarily utilized in the hospital setting. Oral and intranasal forms have been shown to be equally effective [9]. Based on the available drug resource guides and limited literature on this topic, there are no reports of an interaction between oral DDAVP and TF [6-8]. The only study evaluating oral DDAVP absorption with oral intake reported a 40% reduction when ingested within 90 minutes of a standard meal; however, no effect on its antidiuretic effect was reported because it still elicited a maximal response. However, the maximal plasma desmopressin concentration, the time at which the maximal plasma desmopressin concentration was reached, and the time at which plasma desmopressin was first detected were all delayed compared to when oral DDAVP was administered in a fasting state [6]. Thus, gastrointestinal absorption of desmopressin was both reduced and delayed when administered with food [6]. This could be due to either decreased gastrointestinal desmopressin uptake in the presence of food or desmopressin adsorption to food or a combination of both [6]. In addition, this study concluded that the antidiuretic action of desmopressin was not affected despite these pharmacodynamic consequences, based on the data collected for urine volume and urine osmolality [6]. It is important to note that the data were only collected for up to six hours after the administration of a desmopressin dose.

While there is no data on an average known time frame between missing one dose of oral DDAVP and development of signs and symptoms of AVP-D, most pharmacy resources including Medline [8] mention that in case of a missed oral DDAVP dose, one should take it as soon as one remembers but if it is almost time for the next dose, then one should skip it and take the next dose as scheduled. More studies are needed to elucidate how soon AVP-D can develop after missing one dose of oral DDAVP. A case report detailed the development of precipitous arginine vasopressin crisis on withholding an oral desmopressin dose for more than 24 hours [10].

No studies have evaluated oral DDAVP absorption in relation to TF. The Handbook of Drug Administration via Enteral Feeding Tubes mentions DDAVP but indicates that no interactions are noted. This becomes consequential in the vulnerable population of patients who are already receiving TF, and in whom a majority have a concomitantly decreased sense of thirst, preventing intake of free water to restore balance.

## Conclusions

Moreover, one can infer that the absorption of oral DDAVP with concurrent continuous TF administration would be even lower than absorption with one singular meal. We want to draw attention to this phenomenon and note that it warrants further investigation; if it holds, then inclusion of a warning on UpToDate, Micromedex, and Lexicom should be considered. Until further studies are conducted, we recommend switching to subcutaneous dosing in hospitalized patients on TF, especially those with adipsia. Alternative solutions such as increasing the oral DDAVP dose and separating oral DDAVP from TF in time, as with levothyroxine, should also be investigated.
